# Seeing What I Did (Not): Cerebral and Behavioral Effects of Agency and Perspective on Episodic Memory Re-activation

**DOI:** 10.3389/fnbeh.2021.793115

**Published:** 2022-01-07

**Authors:** Benjamin Jainta, Sophie Siestrup, Nadiya El-Sourani, Ima Trempler, Moritz F. Wurm, Markus Werning, Sen Cheng, Ricarda I. Schubotz

**Affiliations:** ^1^Department of Psychology, University of Münster, Münster, Germany; ^2^Otto Creutzfeldt Center for Cognitive and Behavioral Neuroscience, University of Münster, Münster, Germany; ^3^Center for Mind/Brain Sciences, University of Trento, Rovereto, Italy; ^4^Department of Philosophy, Ruhr University Bochum, Bochum, Germany; ^5^Institute for Neural Computation, Ruhr University Bochum, Bochum, Germany

**Keywords:** fMRI, episodic memory, perspective, agency, expectation violation, action observation, action imitation

## Abstract

Intuitively, we assume that we remember episodes better when we actively participated in them and were not mere observers. Independently of this, we can recall episodes from either the first-person perspective (1pp) or the third-person perspective (3pp). In this functional magnetic resonance imaging (fMRI) study, we tested whether agency and perspective modulate neural activity during memory retrieval and subsequently enhance memory performance. Subjects encoded a set of different episodes by either imitating or only observing videos that showed short toy stories. A week later, we conducted fMRI and cued episodic retrieval by presenting the original videos, or slightly modified versions thereof, from 1pp or from 3pp. The hippocampal formation was sensitive to self-performed vs. only observed actions only when there was an episodic mismatch. In a post-fMRI memory test a history of self-performance did not improve behavioral memory performance. However, modified videos were often (falsely) accepted as showing truly experienced episodes when: (i) they were already presented in this modified version during fMRI or (ii) they were presented in their original form during fMRI but from 3pp. While the overall effect of modification was strong, the effects of perspective and agency were more subtle. Together, our findings demonstrate that self-performance and self-perspective modulate the strength of a memory trace in different ways. Even when memory performance remains the same for different agentive states, the brain is capable of detecting mismatching information. Re-experiencing the latter impairs memory performance as well as retrieving encoded episodes from 3pp.

## Introduction

Episodic memories enable us to retrieve information about events from our personal past, including when and where they were experienced (Tulving, 2002). Disturbingly, we have learned that episodic memories are prone to change and decay (Roediger and Butler, 2011; Nader, 2015; Lee et al., 2017). This slow and progressive modification process is presumably fueled by retrieval (Roediger and Butler, 2011; Nader, 2015; Lee et al., 2017), meaning that each time we retrieve an episode, it may undergo subtle revision.

At first glance, episodic memory modification appears to result from some imperfection of the conservative mechanisms our nervous system is equipped with. However, modification of episodic memory may indeed be functional. A fundamental role of episodic memory is the imagination and prediction of the potential future, coined *mental time travel* (Tulving, 2002). It is suggested that internal models derived from our long-term memories inform future choices and behaviors based on previous experiences. Nevertheless, in an ever-changing world, a prerequisite to maintain the efficiency and validity of such internal models is to open them for the integration of new experiences. This updating process is initiated by prediction errors (Exton-McGuinness et al., 2015; Fernández et al., 2016). Accordingly, episodic memories are not only exploited in the course of envisaging the future but are gradually updated by matching them to the current experiences when retrieved.

A major question that derives from this is which conditions render the memory of a truly experienced episode more or less susceptible to later modification. According to recent studies, some subtle breaches of expectation during re-experiencing the original episode is deemed a trigger for such modifications (Sinclair and Barense, 2019). In the current fMRI study, we took advantage of this effect of prediction errors to investigate the influence of two factors on the susceptibility of episodic memory. We employed videos of original episodes that participants had experienced in the lab, and introduced subtle breaches of expectation by modifying either a detail of content (substituting an object) or a detail of structure (swapping two adjacent action steps) in a subset of these videos. We presented both original and modified videos repeatedly during the fMRI session. This was followed by a memory test that assessed memory performance as an indicator of potential episodic updating. Here, we manipulated two factors that we hypothesized would influence an episode’s susceptibility to change:

First, we reasoned that episodes in which we were agents are less prone to modification than those in which we were only observers (factor AGENCY). It was found that self-performed episodes are remembered better than only observed ones (Hornstein and Mulligan, 2001). Moreover, event-related brain potentials differentiate between the re-activation of performed and only observed actions (Senkfor et al., 2002; Leynes and Kakadia, 2013).

Second, we considered that cueing the re-activation of an episode from the first-person or “field” perspective provides a more powerful and vivid re-experience of the original episode than cueing from the third-person or “observer” perspective (factor PERSPECTIVE). It has been reported that episodes remembered (primarily) from the first-person perspective are recalled more accurately and vividly when compared to those (primarily) retrieved from a third-person perspective (Rice and Rubin, 2009; Marcotti and St Jacques, 2018). Also, adopting a first-person perspective as compared to a third-person perspective during retrieval was found to increase activity in the amygdala, signaling greater subjective emotionality (Eich et al., 2009). Yet, another fMRI study reported significant brain activity for third versus first-person perspective retrieval, but no significant clusters for the opposite contrast (Grol et al., 2017).

Thus, self-performance and self-perspective were expected to hamper modification of the original episode, manifesting in high correct rejection scores in the MRI memory test, and a stable surprise-related BOLD response to the manipulated episode videos. In order to test these hypotheses, participants were filmed during encoding of episodic events while either imitating or merely observing PLAYMOBIL^®^-based action stories which were presented in original or modified versions during fMRI in two different perspectives. As we typically experience the world from 1pp, participants are expected to experience this perspective as more persuasive with regard to the representation of themselves in the videos. Thus, by reactivating these episodes and violating participants’ expectations during fMRI we aimed at triggering internal model updating and manipulating memory performance.

In line with prior research (Schiffer et al., 2012, 2013; Siestrup et al., 2021), modified episode videos were expected to trigger substantial brain responses. Therefore, we expected these responses to be stronger for formerly self-performed than for merely observed episodes (H1a), and for videos presenting episodes from the first-person as compared to the third-person perspective (H1b). As to the specific network we expected for the violation response, our hypotheses were mainly focused on the hippocampal formation according to its key role in the re-activation of episodic memories (Rugg and Vilberg, 2013; Jeong et al., 2015). The hippocampal formation is taken to contribute to associative learning, the detection of associative mismatches and the generation of associative predictions (Kumaran and Maguire, 2006; Chen et al., 2011). Moreover, we may see activity in the medial frontal cortex (MFC) which was found to be involved in the processing of competing information for existing episodes, i.e., updating an internal model through new information in order to ensure predictive success (Schiffer et al., 2012, 2013).

With regard to specific behavioral hypotheses, we expected that episodic memories were updated due to repeated presentations of manipulated videos, and accordingly, they should be misclassified as originally experienced during episodic encoding (H2). This hypothesis has been addressed in detail in Siestrup et al. (2021). Moreover, false acceptance of modified videos should be higher for videos that show previously only observed episodes (H3a) and for videos that showed episodes from the third-person perspective (H3b). To rule out the possibility that effects were due to a generalized acceptance bias, we additionally presented entirely new episode videos (“novels”) that we expected participants to classify as new (H4). Regarding reaction time (RT), we did not have any *a priori* hypotheses. Nevertheless, RT has been used previously as an indicator for the length of task-related cognitive processing (Barber et al., 2016). Further, longer RTs were suggested to represent the costs of higher demands in cognitive processing during retrieval (Noppeney and Price, 2004). Therefore, we decided to investigate RTs exploratorily.

## Materials and Methods

### Participants

Forty female right-handed volunteers were recruited to participate in two training sessions and one fMRI scan. Two participants completed the training but did not return for the fMRI session. Additionally, two participants had to be excluded from the analyses of fMRI data due to technical difficulties during the acquisition of functional data or dizziness and nausea during the fMRI session. Of the 36 participants included in the statistical analysis (*M* = 22.67, *SD* = 2.40 years old; range 18–28 years), none reported a history of neurological or psychiatric disorders, or substances abuse. One additional participant had to be excluded for the analysis of the post-fMRI memory test due to misunderstood instructions. As the presented videos showed an actress, only females participated to ensure high self-identification with the stimulus material. The Edinburgh Handedness Inventory was used to assess (Oldfield, 1971) handedness. Participants were all right-handed and scores varied from +60 to +100 (*M* = 92.17, *SD* = 10.95). All participants had normal or corrected-to-normal vision. The study protocol was conducted in accordance with ethical standards of the Declaration of Helsinki and approved by the Local Ethics Committee of the University of Münster. Each participant signed an informed consent and received either reimbursement or course credits for their participation.

### Stimuli

Stimuli were comprised of 76 video clips (mean duration = 12.68 s, range 8.80–17.88 s) showing abstract, but complex stories played with PLAYMOBIL^®^ toys to ensure encoding of unique episodes during training sessions. In each video, an actress was performing while wearing a black pullover and black rubber gloves. The back of the right glove was marked with a yellow dot to ease future imitation (Franz et al., 2007) from demo videos during training. Videos showed only the hands and forearms of the actress and toys, such as animals, characters, furniture, vehicles and tools. Each exact object was only used in one of the stories, which consisted of six to nine action steps (*M* = 7.36) and four to 14 separable objects (*M* = 7).

**FIGURE 1:**
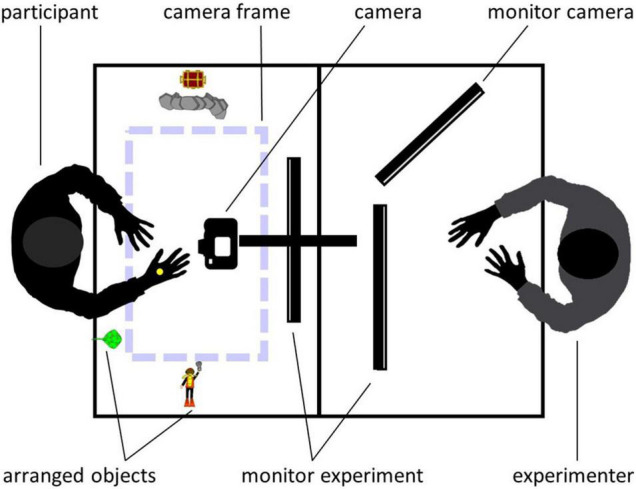
Schematic overview of the filming setup during training sessions. Objects for each story were placed around the camera section (dashed lines). Trials were presented simultaneously for both, experimenter and participant. The participant’s imitation attempts were filmed using the above-mounted camera. The experimenter watched the participant’s performance during imitation trials on an additional screen.

The toy-based stories were filmed on a matte white paper background using a digital reflex camera (Nikon D5300) which was centered above the table and faced straight down. Congruent with the area captured by the camera, a frame (47.5 × 28 cm) was taped on the paper background to visually enclose the camera section for the actress. Every single object needed to replay the story was placed next to the camera section. For a schematic overview of the filming setup, see Figure 1. Videos had a resolution of 1920 × 1080 px and 25 frames per second. All videos were edited with Adobe Premiere Pro CC (Adobe Systems Software, Dublin, Ireland, Version 12.1.2) so that each video started and ended with seven frames showing only background. Original filming perspective was the third-person (or observer) perspective (3pp). In order to create the first-person (or field) perspective (1pp), videos were rotated by 180° as done in a previous study (Wurm and Schubotz, 2018), allowing us to establish the factor PERSPECTIVE during the fMRI session.

A total set of 30 stories was examined in two preceding pilot studies (A and B) to investigate the difficulty of (1) imitating (based on the number of attempts needed to correctly imitate the story three times), (2) interpreting (based on the number of attempts and errors in description), and (3) identifying the story as part of training. Based on this screening, six stories were excluded.

**FIGURE 2:**
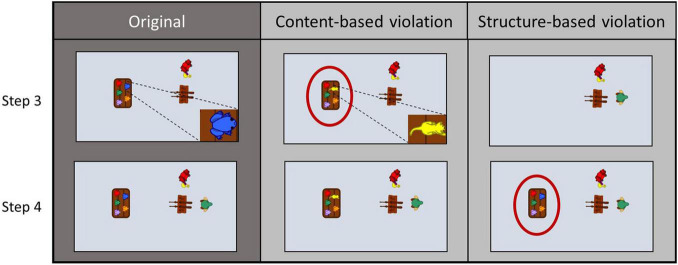
Three different versions of two adjacent action steps for the story “shooting gallery”. Sample video frames of the original version **(left column)** show how a table with five different colored frogs is placed (step 3) before the fair visitor with the green hat enters the scene (step 4). In the content-based modification **(middle column)** the blue frog on the table is replaced by a yellow cat, while in the structure-based modification **(right column)** the table with the five different colored frogs is placed (step 4) only after the fair visitor entered the scene (step 3).

Of the remaining 24 stories, there were three different versions of each, one original and two modified ones. The original versions were presented to participants, imitated or observed and described only. The modified versions involved either a structure-based modification (*str*), in which two adjacent action steps were swapped to elicit a sequential surprise, or a content-based modification (*con*), in which an object was swapped to elicit an object-semantic surprise (see Figure 2 for an illustration). Effects regarding the factor modification (str, con) are only addressed in a companion paper (Siestrup et al., 2021). Note that the factors addressed in this paper were statistically independent of those reported in the mentioned paper. Modifications in the videos never occurred in the first two or last two actions steps of a story. Furthermore, four stories were additionally presented in only one version each during the fMRI session, which served as a control condition (hereafter referred to as “novels”).

### Training Procedure

The training consisted of two sessions (approximately 2.0 and 1.5 h) on two consecutive days. In order to avoid fatigue or motivational decrease due to the long duration of the task, we chose to split the training over two consecutive days. During training, participants imitated half of the 24 stories from the original video clips and solely observed the other half. Each training session consisted of an imitation block and an observation block. Additionally, the first session included a short practice to get used to observing, imitating, and describing the action videos.

**FIGURE 3:**
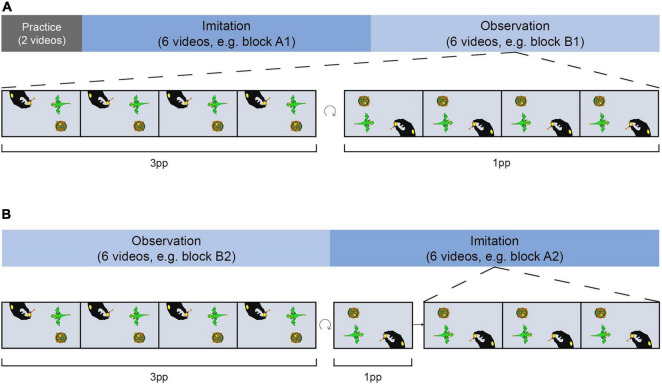
Training procedure. Training took place on two consecutive days. Half of the actions were imitated and the other half were only observed by participants. Imitation and observation videos were presented within blocks of six videos each. **(A)** After a short practice on the first day, participants either started the training with an imitation block on the first **(A)** and an observation block on the second day **(B)** or vice versa. In the imitation block, each demo video was presented four times from 3pp and one time from 1pp before participants had to accurately imitate each action three times. During observation blocks, participants were presented with demo videos four times from 3pp and four times from 1pp. At the end of each trial, participants had to provide a detailed description of the action story.

Twenty four stories were organized in four blocks (A1, B1, A2, B2; see Figure 3), each consisting of six videos and balanced for the number of actions steps. The assignment of videos to blocks remained the same while the running order of the videos was randomized in each block. Participants either imitated videos of blocks A1 and A2 and only observed videos of blocks B1 and B2 or vice versa, implementing the factor *AGENCY (imi, obs)*. The blocks and block order were balanced among participants. Thus, all actions were equally often imitated or only observed in the course of the study. Further, half of the participants started with an imitation block while the other half started with an observation block after the practice phase, and vice versa on the second training day.

To determine the number of video presentations needed to correctly imitate an action, we conducted two pilot studies. When participants were free to choose the number of video presentations (pilot study A), they watched a demo video a median of four times. In addition, we controlled whether the chosen number was suitable to imitate the actions from our set of stories (pilot study B). Based on our pilot data, subjects were presented with each demo video five times during the imitation blocks of the training session (four times from 3pp, once from 1pp). To ensure accurate encoding of the stories, participants had to correctly imitate each action three times. To counterbalance the different perspective experiences during the imitation and observation blocks, demo videos were presented in the observation blocks from both perspectives (four times from 3pp and four times from 1pp). With respect to the recognizability of the actions, the toys for each action in each block were arranged around the camera section (Figure 1) in the same way as when original versions were created.

During the imitation blocks, participants were asked to imitate actions as accurately as possible with regard to object orientation, speed, hand position etc. To ensure accurate encoding of the episodes, only trials without errors were classified as “successful attempt.” If participants made a mistake, the experimenter interrupted them immediately and subjects had to rearrange the objects around the camera section and start again. The number of imitation attempts was not limited.

After subjects had imitated or merely observed an action, they were asked to give a detailed description of the story. A successful description included all the action steps in the correct order, including all objects that appeared in the correct color and the actions performed by the characters in the story. In this way, we ensured that the participants were attentive and understood the story correctly. If a participant made a mistake during the description, the experimenter interrupted them immediately and pointed out the error. The participants where then asked to start describing the current story again. The number of attempts to describe a story was not limited.

#### Cover Story

Participants were told that they would be filmed during action execution and some of these videos would be presented during the fMRI experiment. This cover story was used to ensure that, maintaining a high level of standardization of the stimuli, there was still a personal identification with the actress in the videos.

To test the cover story, we tested participants’ identification with the actor in the videos in another pilot study (B) one week after the training. To do so, we asked 16 independent participants to rate on a scale from 1 (“no”) to 4 (“yes”) whether they believed they appeared in the presented video (“Was this you in the video?”). A repeated measures ANOVA (rmANOVA) revealed a significant main effect of AGENCY [*F*_(1_,_15)_ = 33.130, *p* < 0.001]: Stronger identification was found for videos that participants had previously imitated (*M* = 2.89 ± 0.11) (mean ± standard error of mean) than for those that had previously observed only (*M* = 2.12 ± 0.15). Additionally, participants indicated that 45% of the videos showed themselves (*M* = 45% ± 2.28%, Range = 20–80%). As a reminder, although they were filmed during training, none of the videos showed the participants; it was always the same actress. These results suggest that participants believed the cover story and were largely convinced that they were seeing videos of themselves during testing.

### Functional Magnetic Resonance Imaging Session

The fMRI experiment lasted approximately 50 min. Participants had already practiced the task briefly at the end of the second training session, and the practice trials were not used again in the fMRI session. Participants were presented with original and modified videos of the previously encoded stories. Each participant saw only either the original (*ori*) or the modified (*str*/*con*) version of a story. Out of the 24 stories encoded during training session, eight videos were presented in the original, eight in a structure modified and eight in a content modified version. Stories were assigned to each condition in a counterbalanced manner between participants, i.e., each video was presented equally often in each condition. With regard to the training procedure, half of the presented videos during fMRI showed stories that were previously imitated (imi) while the other half showed only observed videos (obs). The videos were presented either from the 1pp or from the 3pp, establishing the factor PERSPECTIVE. The assignment of videos to retrieval cueing perspective was counterbalanced among participants. We thereby obtained a 3 × 2 × 2 within-subject design with twelve experimental conditions, i.e., MODIFICATION (ori, str, con) × AGENCY (imi, obs) × PERSPECTIVE (1pp, 3pp). Please note that we aggregated content- and structure-based conditions which together included double as many trials as original versions and will be referred to as modified versions (mod) in the following sections. In each of the resulting 12 factorial combinations, two stories were presented six times each. The two stories contained actions of which one had been encoded during the first and the other one during the second training session. Four novel actions were included in the fMRI session to serve as a control condition, of which two were presented from 1pp and two from 3pp.

**FIGURE 4:**
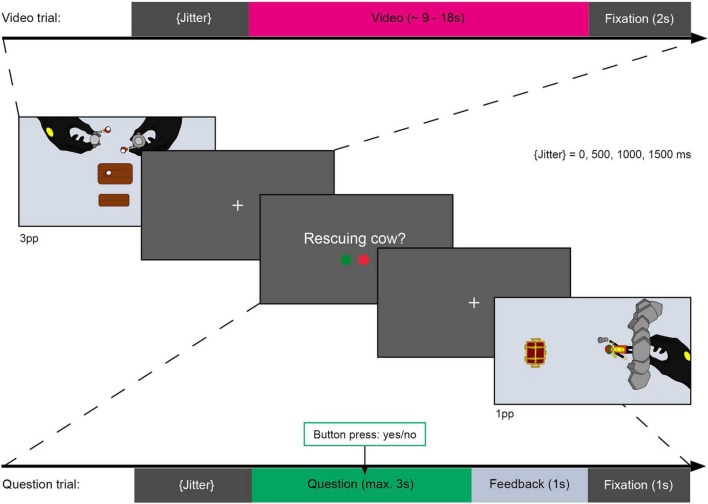
Schematic illustration of task during fMRI session. Top bar showing duration and sequence of a video trial. Video trials contained a variable jitter (0, 500, 1000, or 1500 ms of fixation), a video showing a PLAYMOBIL^®^ story (approx. 9 – 18 s) and a second fixation cross (2 s) serving as an interstimulus interval (ISI). Video trials were presented from an allocentric perspective (3pp, first image) or an egocentric perspective (1pp, last image). After some of the videos, a question regarding the action shown in the preceding video was presented. Question trials consisted of a variable jitter, a question presentation (max. 3 s or terminated by response) and an ISI, containing a 1 s feedback presentation (“correct,” “incorrect,” or “too slow”) and a 1 s fixation cross. Participants had to respond by accepting (right index finger) or rejecting (right middle finger) the presented action description via a response box.

After 38.89% of the videos, a short description was presented (see Figure 4) that either matched or did not match the content of the preceding video (question trials). These question trials were used to ensure that participants attentively watched and recognized the action videos as shown in a previous study (El-Sourani et al., 2018). Accordingly, participants had to either accept or reject the description using two predetermined buttons on a response box. Questions were presented for a maximum of 3 s or until participants responded. Participants received written feedback (correct/incorrect/too late) after each question. During the experiment, each video was shown once with a matching description and once with a non-matching description.

The fMRI experiment comprised 242 trials divided into 6 blocks with 40 to 41 trials each. Each of these blocks contained 24 videos of previously encoded actions, three null events in which only a fixation cross was presented for 7–10 s, and 9–10 questions, of which approximately half were to be accepted and the other half rejected. In addition, each of the four novel videos was presented once per block. Thus, the entire experiment contained 144 video trials of previously encountered episodes, 18 null events, 56 question trials, and 24 novel video trials. Trials were variably jittered (0, 500, 1000 or 1500 ms) and ended with a fixation cross (2 s after videos or 1 s after questions). The trial order was pseudorandomized to balance the transition probabilities between conditions and the order of presentation of the conditions within each block. A maximum of four video trials were presented consecutively throughout the experiment.

#### Functional Magnetic Resonance Imaging Acquisition

Imaging was performed on a 3-Tesla Siemens Magnetom Prisma MR tomograph using a 20-channel head coil and took place approximately one week after the second session (*M* = 7.42 days, *SD* = 0.9 days). Participants were located in a supine position on the scanner bed with their right index and middle finger positioned on two predetermined response buttons on a response box. To minimize arm and head motions, arms and heads were tightly fixated with form-fitting cushions. Additionally, participants were provided with earplugs and headphones to attenuate scanner noise. Stimulus presentation and response coding were performed using Presentation 20.3 (Neurobehavioral Systems, San Francisco, CA, United States). Stimuli were projected onto a screen at the end of the scanner bore. Participants saw the screen on an individually adjusted mirror mounted to the head coil.

Prior to functional imaging, high resolution T1 weighted anatomical images were obtained with a 3D-multiplanar rapidly acquired gradient-echo (MPRAGE) sequence. 192 slices with a thickness of 1 mm were acquired, using a repetition time (TR) of 2130 ms, an echo time (TE) of 2.28 ms, a flip angle of 8° and a field of view (FoV) of 256 × 256 mm^2^. Functional images of the whole brain were acquired in interleaved order along the bicomissural plane (AC–PC) using a gradient-echo echoplanar imaging (EPI) sequence sensitive to BOLD contrast. Thirty-three axial slices with a thickness of 3 mm were obtained in an interleaved order, using a TR of 2000 ms, a TE of 30 ms, a FoV of 192 × 192 mm^2^ and a flip angle of 90°.

Imaging data were processed using SPM12 (Wellcome, Trust, London, United Kingdom) implemented in MATLAB R2018b. First, slice time correction to the middle slice was performed, followed by movement correction and realignment to the mean image. Then, individual structural scan was co-registered to the mean functional image and segmented into native tissue components. Functional and structural images were normalized into the standard MNI space (Montreal neurological Institute, Montreal, QC, Canada). Spatial smoothing was based on a Gaussian kernel of full-width at half maximum (FWHM) of 8 mm. Additionally, a 128 s high-pass temporal filter was applied.

#### Functional Magnetic Resonance Imaging Design Specifications

The statistical analysis of the fMRI data was based on a least-squares estimation using the general linear model (GLM) for serially autocorrelated observations (Friston et al., 1994; Worsley and Friston, 1995). The GLM convolved regressors with a canonical hemodynamic response function and contained a total of 18 regressors: eight predictors for the experimental conditions, one predictor for null events, one for question trials, two for novel videos, and six regressors of nuisance for the motion parameters (three translations, three rotations). Video trials were assigned to the eight experimental condition regressors with regard to whether they (1) showed an original (*ori*) or a modified version (*mod*) of previously (2) imitated (*imi*) or only observed (*obs*) action stories (3) presented in a 1pp or a 3pp. Activations were analyzed time-locked to the onset of the videos and the analyzed epoch comprised the full duration of the presented videos (8.80–17.88 s). The modeled activation of null events (7–10 s) and questions (max. 3 s) was time-locked to their respective onsets. Question trials were modeled as events. Novel videos (nov) were assigned to two regressors regarding their stimulus-presentation mode (1pp vs. 3pp).

On the first level of the analysis, we applied gray matter masking. Here, we used smoothed individual normalized gray matter images (8 mm FWHM) thresholded at 0.2 using ImCalc in SPM12 creating a binary mask. On the second level, we performed group analyses by using one-sample *t*-tests across participants. A false discovery rate (FDR) correction with a threshold of *p* < 0.05 or higher (peak level) was applied. When no significant activation clusters were found using this threshold, we applied a threshold of *p* < 0.001, uncorrected for multiple comparisons. This is a common approach when specific neuroanatomic hypotheses are investigated (Farrer and Frith, 2002; Mechelli et al., 2006; Kumaran and Maguire, 2007).

To investigate whether brain activity differs for retrieved action episodes compared to novel stories, we calculated first-level *t* contrasts for ori > nov and mod > nov. Because original episodes as well as the slightly modified episodes should lead to the retrieval of episodic memories in contrast to previously unencoded episodes (novels), we built the conjunction of these contrasts. This approach was used to provide (i) the validation for successful retrieval of encoded episodes and (ii) the basis that effects of AGENCY and/or PERSPECTIVE underlie episodic memory retrieval.

To test the effects of perspective, we built separate contrasts of 1pp > 3pp and 3pp > 1pp for original and modified episodes. Then, we analyzed whether perspective had a specific effect on episodic retrieval as compared to novel videos. To this end, we also calculated the 1pp vs. 3pp contrast for novel videos and considered the interaction. Confounding effects of modification on perspective were ruled out by building conjunctions over perspective contrasts of original and modified episodes. To test the effects of agency, we obtained a conjunction for imitated vs. only observed original and modified episodes to analyze the impact of agency (ori_imi_ > ori_obs_ ∩ mod_imi_ > mod_obs_).

To explore hippocampal activity for original vs. modified episodes that have either been imitated or only observed during training, we conducted ROI analyses for left and right hippocampus by extracting beta values for the regressors ori_imi_, ori_obs_, mod_imi_, and mod_obs_. Mean beta values for each regressor were extracted using the MarsBaR Toolbox (Brett et al., 2002). For statistical analysis, we used a three-way rmANOVA with the factors HEMISPHERE (left, right), MODIFICATION (ori, mod) and AGENCY (imi, obs) and post-hoc pairwise *t*-tests for ori_imi_ vs. ori_obs_ and mod_imi_ vs. mod_obs_. Here, we were specifically interested in the effects of agency on hippocampal activity. Therefore, we additionally performed two paired *t*-tests (one-tailed) for the comparisons of ori_imi_ > ori_obs_ and mod_imi_ > mod_obs_ in each hemisphere separately. We applied a significance level of α = 0.05, Bonferroni–Holm-adjusted for multiple comparisons (Holm, 1979). To this end, anatomical ROIs of the left and right hippocampus (including the CA1, CA2, CA3, dentate gyrus, subiculum, entorhinal cortex and the hippocampal-amygdaloid transition region) were created from probabilistic maps from the Julich-Brain Cytoarchitectonic Atlas (Amunts et al., 2020). A threshold of 0.2 was implemented in ImCalc and final ROIs were created using the MarsBaR toolbox (Brett et al., 2002) in SPM12. Further, we used Pearson’s correlation coefficient to examine the relationships between mean beta values and behavioral memory performance for original and modified videos separated by MODIFICATION during fMRI and AGENCY.

### Memory Test

After the fMRI experiment, participants conducted a memory test which took approximately 15 min. In a separate room, subjects were instructed to watch action videos on a laptop and to rate whether they remember the exact story presented from the training session. Responses were measured on a four-point Likert scale (1: *yes*; 2: *rather yes*; 3: *rather no*; 4: *no*) by pressing one of four marked keys on the laptop’s keyboard. There was no time restriction for responses, but extreme outliers were removed as described in the following section.

Participants were presented with two versions of each of the stories they had seen during fMRI, an original and a modified version. If they had seen a modified version during fMRI, they were now presented with the same modified version as well as the corresponding original version. If participants had seen the original version during the fMRI, they now saw a corresponding modified version in addition to the original. Thus, responses were always given for an original and a modified video of the exact same story that had been seen for the first-time during training. Each novel video was presented twice. Thus, participants had to respond to a total of 56 videos. These responses indicated how well the participants could remember the individual storylines.

### Behavioral Data Analysis

Behavioral data from the fMRI session and the post fMRI memory test were analyzed using RStudio (R Core Team, 2019; version 1.2.5001).

Performance during the fMRI session was assessed by correct response rates and RTs on correctly answered question trials.

For the analysis of the memory test, we used participants’ mean rating times, i.e., RTs of correct and incorrect responses, and mean ratings on remembering a presented episode from the training on a four-point Likert scale. Please note that high ratings mean low acceptance, while low ratings mean high acceptance. After completing the memory test, one participant reported difficulties in understanding the task correctly. Thus, we excluded data from this subject from the behavioral analysis of the memory test. Considering rating times during the memory test, a single trial was excluded in advance as one participant left the laptop to talk to the experimenter.

Data distribution was tested by using the Shapiro–Wilk Test. When RTs and rating times did not fit normal distribution, we applied logarithmic transformation to make data conform to normality in order to use parametric rmANOVA. As ratings were not normally distributed, we used a non-parametric rmANOVA based on aligned rank data (Wobbrock et al., 2011). For parametric and non-parametric rmANOVAs, we used a 2 × 2 × 2 within-subject design with the factors MODIFICATION (ori, mod) during fMRI, encoding AGENCY (imi, obs) and PERSPECTIVE (1pp, 3pp). Please note, that we aggregated modified videos (str, con) for the analysis of behavioral responses as the main focus of this study was to investigate the effects of agency and perspective on true and false episodic memories. For a more detailed analysis of the modification conditions (con, str), see Siestrup et al. (2021). We separately investigated mean ratings and rating times for original and modified videos during memory test in order to generate higher discriminatory power for subtle effects on true and false memories. *Post hoc* pair-wise comparisons were conducted with paired *t-*tests (one-tailed). With regard to our control condition of novel stories, we used paired sample *t*-tests (two-tailed).

The significance level for all behavioral analyses was set to *p* < 0.05. In order to compensate for multiple comparisons, *p*-values were adjusted using the Bonferroni–Holm correction (Holm, 1979).

## Results

### Behavioral Results of the Functional Magnetic Resonance Imaging Session

During the fMRI experiment, participants rejected short descriptions of each preceding video as inaccurate or accepted them as accurate by selecting the corresponding response button. As subjects very rarely responded incorrect to questions during fMRI, we did not find any significant effect in correct response rates. Thus, we only report descriptive values. Participants correctly answered 98.2% ± 0.55% (mean ± standard error of mean) of question trials following an original video and 98.3% ± 0.38% following a modified video. For our control condition, participants correctly answered 96.8% ± 1.04% of question trials following a new video. Bar charts of the three-factor design regarding the correct response rates are provided in Supplementary Figure 1.

With regard to RT on correct trials, a three-way rmANOVA with the factors story MODIFICATION (original, modified), stimulus presentation mode PERSPECTIVE (1pp, 3pp) and pre-fMRI training mode AGENCY (imitated, observed) revealed a significant main effect for the factor PERSPECTIVE [*F*_(1_,_35)_ = 6.39, *p* = 0.02]. Thus, subjects were significantly faster when presented with a video from 1pp (*M_1*pp*_* = 958.66 ms ± 20.12 ms) than from 3pp (*M_3*pp*_* = 980.06 ms ± 20.63 ms). There were no significant main effects for the factors MODIFICATION (*M*_*ori*_ = 963.72 ms ± 21.68 ms; *M*_*mod*_ = 975 ms ± 19.02 ms) or AGENCY (*M*_*imi*_ = 977.05 ms ± 20.92 ms; *M*_*obs*_ = 961.68 ms ± 19.85 ms).

**FIGURE 5:**
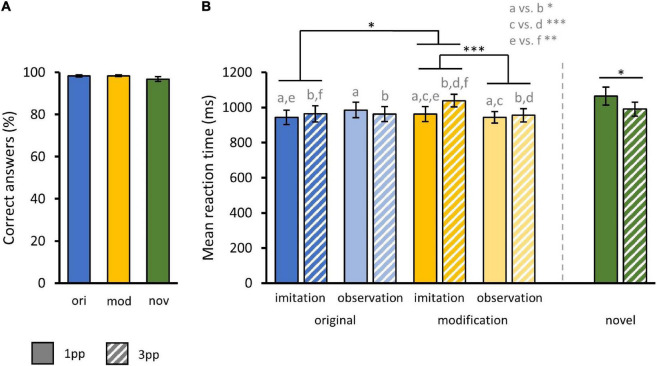
Behavioral results from fMRI task. **(A)** Mean correct response rates for original (ori), modified (mod), and novel (nov) videos during fMRI action recognition task. **(B)** Mean reaction times for original and modified videos separated by the factors AGENCY (imitation, observation) and PERSPECTIVE (1pp, 3pp) and additionally for novels. Statistics: rmANOVA with *post hoc* paired *t*-tests; a vs. b = *p* < 0.05; c vs. d = *p* < 0.001; e vs. f = *p* < 0.01. **(A,B)** Bar charts show means and standard errors.

In addition, we found a significant interaction effect of MODIFICATION and AGENCY [*F*_(1_,_35)_ = 4.83, *p* = 0.03]. Paired samples *t*-tests showed that participants were significantly slower when previously imitated episodes were presented in a modified (*M_*mod*–*imi*_* = 1001 ms ± 38.16 ms) compared to an original version [*M_*ori*–*imi*_* = 953.86 ms ± 41.15 ms; *t*_(35)_ = 2.39, *p* = 0.01] but also when compared to a modified version of an episode that had only been observed before [*M_*mod*–*obs*_* = 949.64 ms ± 34.86 ms; *t*_(35)_ = 4.14, *p* < 0.001]. Accordingly, subjects took longer to recognize modified videos when these videos showed previously self-enacted stories. Further, we found a significant interaction of MODIFICATION and PERSPECTIVE [*F*_(1_,_35)_ = 8.02, *p* = 0.007]. When presented with a modified video from 1pp (*M_*mod–*1*pp*_* = 952.91 ms ± 27.18 ms) participants were significantly slower [*t*_(35)_ = 3.9, *p* < 0.001] as compared to 3pp videos (*M_*mod–*3*pp*_* = 997.1 ms ± 26.56 ms) while this was not the case for originals [*M_*ori–*1*pp*_* = 964.41 ms ± 29.85 ms, *M_*ori–*3*pp*_* = 963.04 ms ± 31.64 ms; *t*_(35)_ = 0.28]. There was also a significant interaction of AGENCY and PERSPECTIVE [*F*_(1_,_35)_ = 4.88, *p* = 0.03]. Participants were significantly slower when presented with a video showing a previously imitated episode from 1pp vs. 3pp [*M_*imi–*1*pp*_* = 952.83 ms ± 29.64 ms, *M_*imi–*3*pp*_* = 1001.27 ms ± 29.44 ms; *t*_(35)_ = -2.96, *p* < 0.01]. This was not the case for formerly only observed actions [*M_*obs–*1*pp*_* = 964.48 ms ± 27.40 ms, *M_*obs–*3*pp*_* = 958.04 ms ± 28.9 ms; *t*_(35)_ = 0.61, *p* = 0.27]. Finally, participants were significantly slower to respond to new videos from 1pp (*M_*nov–*1*pp*_* = 1064.06 ms ± 52.07 ms) than from 3pp [*M_*nov–*3*pp*_* = 989.69 ms ± 39.53 ms; *t*_(35)_ = 2.34, *p* = 0.01] (Figure 5).

### Functional Magnetic Resonance Imaging Results

#### Main Effect of Episodic Reactivation

**FIGURE 6:**
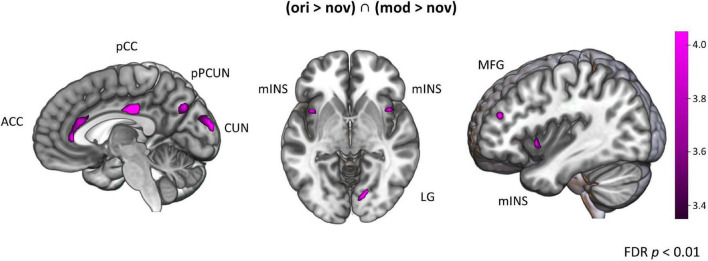
Brain activation for episodes. Areas activated for action videos showing formerly experienced stories contrasted with novel stories [(*ori* > *nov*) ∩ (*mod* > *nov*)]. FDR-corrected *t*-map (*p* < 0.05). CUN, cuneus; PCUN, precuneus; pCC, posterior cingulate cortex; ACC, anterior cingulate cortex; MFG, middle frontal gyrus; mINS, mid-Insula; LG, lingual gyrus.

To investigate which brain regions are generally involved in episodic memory re-activation we contrasted formerly encoded with completely new stories. This was done separately for original (ori > nov) and modified (mod > nov) episodes. We then built the conjunction of these two contrasts (ori > nov ∩ mod > nov) to determine regions that were active when participants retrieved episodic memories, no matter whether encountered in the original or a modified version. Here, we found significant activity in the left posterior mid cingulate cortex (pCC), the left posterior precuneus (pCUN), the right cuneus (CUN), the left anterior cingulate cortex (ACC), the bilateral midfrontal gyrus (MFG), the bilateral mid-Insula and the right lingual gyrus (LG) (Table 1 and Figure 6). The reverse contrast (nov > ori) revealed a widespread activity pattern including increased activity in bilateral hippocampus (HC) (Supplementary Figure 2 and Supplementary Table 1).

**TABLE 1:** Peak activations from second-level whole-brain analyses of episodic effects.

Area	*H*	Cluster extent (voxels)	MNI Coordinates			Z
			*x*	*y*	*z*	
**(*ori* > *nov*) ∩ (*mod* > *nov*)**						
pCC	L	120	–3	–25	35	5.40
CUN	R	209	18	–91	23	5.38
	L		–6	–100	17	4.46
Posterior PCUN	L	59	–9	–67	32	4.12
ACC	L		–3	26	20	3.65
MFG	L	10	–39	44	20	3.94
	R	7	39	44	8	3.80
Insula	L	29	39	11	–7	3.89
	R	18	–36	8	–7	4.13
LG	R	23	15	–76	–7	4.34

*H, hemisphere; L, left; R, right; MNI, Montreal Neurological Institute; PCUN, precuneus; pCC, posterior cingulate cortex; CUN, cuneus; ACC, anterior cingulate cortex; MFG, middle frontal gyrus; LG, lingual gyrus. FDR-corrected at p < 0.05.*

#### Cerebral Effects of Former Self-Performance in Reactivated Episodes

To test the hypothesis that formerly self-performed episodes produce stronger neural activity than merely observed episodes, we first contrasted formerly imitated versus observed episodes for original (ori_imi_ > ori_obs_) and modified videos (mod_imi_ > mod_obs_). While we did not find any effect for the original videos, there was subthreshold activity for the modified episodes contrast (uncorr., *p* < 0.001) in the left hippocampus (HC; *x* = –27, *y* = –22, *z* = –13, *Z* = 3.71), the left posterior PCUN (*x* = –6, *y* = –67, *z* = 23, *Z* = 3.83) and the left MFC in Brodmann area 10 (BA 10; *x* = –12, *y* = 56, *z* = 2, *Z* = 3.59).

**FIGURE 7:**
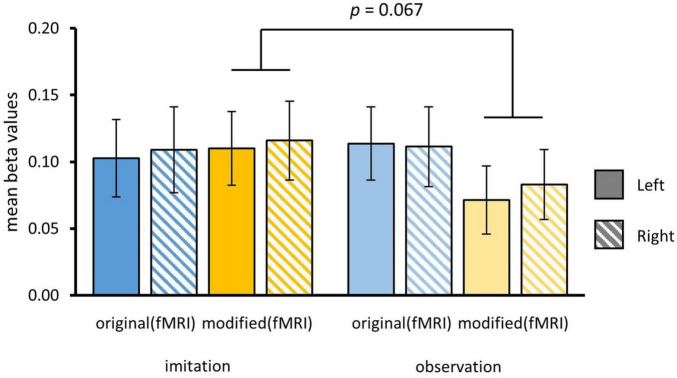
Beta values for ROI of left and right hippocampal complex (HC). Beta values were separately extracted for original and modified videos for actions that have been formerly imitated or only observed. Contrasts were computed for each condition vs. implicit baseline. Bars show means and standard errors of means. Beta values were significantly higher in left hippocampus for imitated than only observed actions during presentation of modified videos.

To further investigate the hypothesis that HC was more strongly involved in episodic memory for imitated vs. only observed episodes, we performed a region of interest (ROI) analysis. ROIs of the right and left HC were created using probabilistic maps from the Julich Brain Cytoarchitectonic Atlas (Amunts et al., 2020). We separately contrasted formerly self-performed and only observed actions for original (ori_imi_, ori_obs_) and modified episodes (mod_imi_, mod_obs_). We used a three-way rmANOVA with the factors HEMISPHERE, MODIFICATION and AGENCY and found a marginally significant interaction effect of MODIFICATION and AGENCY [*F*_(1_,_34)_ = 3.57, *p* = 0.07]. As the whole-brain contrast indicated stronger activation in the left HC, we exploratorily investigated the interaction in both HC for original and modified versions. We did not find any significant main effect or further interactions. For the left HC, paired *t*-tests revealed that observed actions (*M*_*mod–obs*_ = 0.07 ± 0.03) vs. self-performed (*M*_*mod–imi*_ = 0.11 ± 0.03) produced decreased activity in left HC when presented in a modified version [*t*_(35)_ = 2.19, *p* = 0.02], whereas this was not the case for original versions [*M*_*ori–imi*_ = 0.1 ± 0.03, *M*_*ori–obs*_ = 0.11 ± 0.03; *t*_(35)_ = –0.7, *p* = 0.24]. Though descriptively showing a similar tendency, no significant effect was found for beta scores in the right HC ROI (Figure 7).

#### Cerebral Effects of First-Person Perspective During Episode Reactivation

**FIGURE 8:**
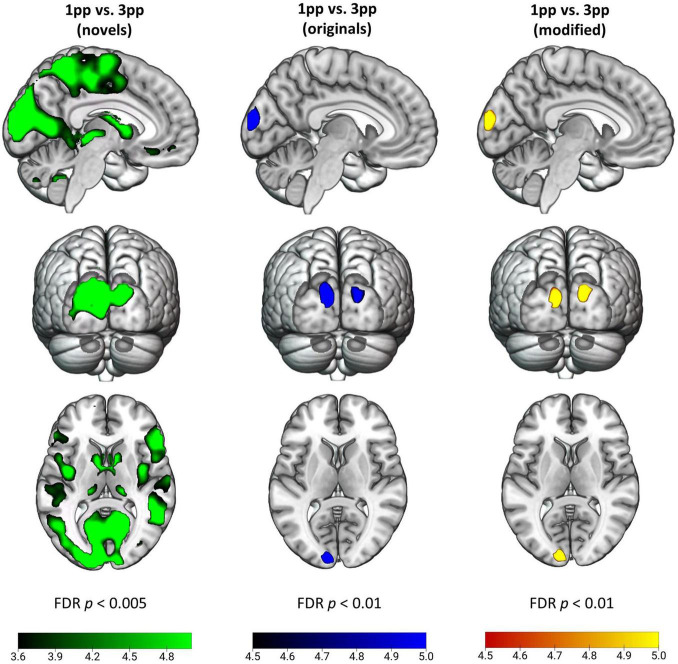
Effects of perspective for novel (green), original (blue), and modified (yellow) stories. Activity patterns indicate enhanced activity for a first-person perspective (1pp) compared to a third-person perspective (3pp). For better visualization we show t-maps at *p* < 0.005 (FDR-corrected) for the novel videos, and *p* < 0.01 (FDR-corrected) for original and modified videos. New videos presented from 1pp led to increased activity in supplementary motor area, thalamus, posterior insula, cuneus, supramarginal gyrus and inferior frontal gyrus among others. Original and modified videos showed more engaged activation in bilateral cuneus.

To test the hypothesis that episodic reactivation from the 1pp elicits a higher BOLD response than reactivation from the 3pp due to more vivid re-experiencing, we contrasted 1pp videos with 3pp videos separately for original and for modified stories. During presentation of an original episode from 1pp (ori_1pp_ > ori_3pp_) we found more pronounced activity in the bilateral CUN. This effect was also found for modified videos (mod_1pp_ > mod_3pp_). For completeness, we also report the effects of reversed contrasts, for which we had no hypotheses: Showing videos of original episodes from 3pp (ori_3pp_ > ori_1pp_) resulted in increased activation in the right LG and the left dorsal PM (PMd), whereas presenting modified videos from 3pp (mod_3pp_ > mod_1pp_) revealed activity in the bilateral inferior parietal lobe, the right LG, the right CUN, the right medial temporal gyrus and the left SMA (Figure 8 and Table 2; FDR-corrected at *p* < 0.05).

**TABLE 2:** Peak activations from second-level whole-brain analyses of perspective effects in episodes.

Area	*H*	Cluster extent (voxels)	MNI Coordinates			*Z*
			*x*	*y*	*z*	
***ori_1*pp*_* > *ori_3*pp*_***						
CUN	L	137	–9	–94	11	6.00
	R	61	18	–91	14	4.07
***ori_3*pp*_* > *ori_1*pp*_***						
LG	R	735	6	–82	–1	INF
PMd	L	13	–51	–13	53	4.07
***mod_1*pp*_* > *mod_3*pp*_***						
CUN	L	109	–9	–94	14	5.52
	R	61	18	–94	23	5.45
***mod_3*pp*_* > *mod_1*pp*_***						
IPL	L	20	–51	–52	53	3.34
	R	25	54	–55	47	3.44
SMA	L	28	–9	26	47	3.60
MTG	R	28	42	–64	11	3.60
CalcS extending into CUN	R	1064	6	–85	–1	7.59
LG	R		15	–73	–7	7.11

*L, left; R, right; MNI, Montreal Neurological Institute; CUN, cuneus; LG, lingual gyrus; PMd, dorsal premotor cortex; IPL, inferior parietal lobe; SMA, supplementary motor area; MTG, medial temporal gyrus; CalcS, calcarine sulcus. FDR-corrected at p < 0.05.*

**FIGURE 9:**
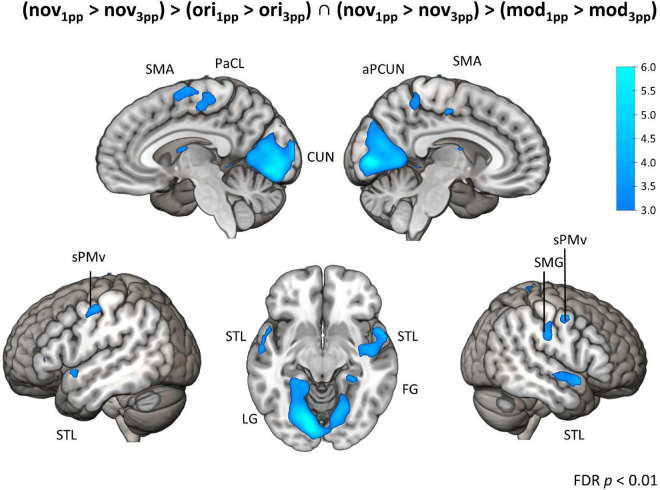
Brain activation for perspective. Conjunction contrast showing enhanced brain activity for interaction contrasts of novels vs. original and novels vs. modified versions (nov_1pp_ > nov_3pp_) > (ori_1pp_ > ori_3pp_) ∩ (nov_1pp_ > nov_3pp_) > (mod_1pp_ > mod_3pp_). Activity patterns indicate enhanced activity for a first-person perspective (1pp) compared to a third-person perspective (3pp) for *t*-map at *p* < 0.01 (FDR-corrected). SMA, supplementary motor area; PaCL, paracentral lobule; aPCUN, anterior precuneus; CUN, cuneus; sPMv, superior ventral premotor cortex; STL, superior temporal lobe; LG, lingual gyrus; FG, fusiform gyrus; PoCG, postcentral gyrus.

In sharp contrast to these moderate effects, we found strong and widespread activity patterns for novel stories presented from 1pp (nov_1pp_ > nov_3pp_). Activation was higher in several areas as e.g., the left supramarginal gyrus (SMG) (*x* = –54, *y* = –25, z = 44, *Z* = 7.67), the right inferior PMd (*x* = 54, *y* = 8, *z* = 29, *Z* = 5.27), the left IFG (*x* = 57, *y* = 32, *z* = 5, *Z* = 5.22). Notably, the reverse comparison (nov_3pp_ > nov_1pp_) did not yield any significant activation. To statistically validate the difference in perspective effects between encoded episodes and novel videos, we built the conjunction of the three contrasts [(nov_1pp_ > nov_3pp_) > (ori_1pp_ > ori_3pp_) ∩ (nov_1pp_ > nov_3pp_) > (mod_1pp_ > mod_3pp_)]. This revealed more pronounced activation for novels in a widespread set of areas, including the bilateral superior ventral premotor cortex (sPMv), anterior precuneus (aPCUN), the bilateral superior PM the right SMG, cuneus (CUN; extending from calcarine sulcus into cuneus), the bilateral superior temporal lobe (STL), the left lingual gyrus (LG) and the right fusiform gyrus (FG) (Table 3 and Figure 9).

**TABLE 3:** Peak activations from second-level whole-brain analyses of perspective effects.

Area	*H*	Cluster extent (voxels)	MNI Coordinates			*Z*
			*x*	*y*	*z*	
**(*nov1pp* > *nov3pp*) > (*ori1pp* > *ori3pp*) ∩ (*nov1pp* > *nov3pp*) > (*mod1pp* > *mod3pp*)**						
Postcentral gyrus	R	12	21	–37	74	3.65
Paracentral lobule	L	1	–9	–28	59	3.32
SMA	L	1	–6	–19	53	3.41
	e. i. R	3	0	–22	65	3.40
aPCUN	R	49	15	–43	50	4.09
Superior ventral PM	L	8	-45	–10	50	3.50
	R	13	48	–1	50	3.66
SMG	R	14	51	–19	32	3.70
CUN	L		–18	–67	–7	4.93
STL	L	1	–57	5	–10	3.55
	R	71	60	5	–10	4.03
LG e. i. FG and PHG	L		–12	–82	–13	5.72
FG e. i. PHG	R	29	27	–34	–16	3.97

*L, left; R, right; e. i., extending into; MNI, Montreal Neurological Institute; SMA, supplementary motor area; aPCUN, anterior precuneus; PM, premotor cortex; SMG, supramarginal gyrus; CUN, cuneus; STL, superior temporal lobe; PHG, parahippocampal gyrus; LG, lingual gyrus; FG, fusiform gyrus. FDR-corrected at p < 0.01.*

### Behavioral Results of the Post-functional Magnetic Resonance Imaging Memory Test

We analyzed memory performance by averaging responses to original_MT_ and modified_MT_ videos separately. We used two three-way rmANOVA based on aligned ranks with the factor stimulus MODIFICATION_fMRI_ (original_fMRI_, modified_fMRI_), presentation mode PERSPECTIVE (1pp, 3pp) and pre-fMRI training mode AGENCY (imitated, observed).

First, we tested whether original episodes were rejected more often after repeated presentation in a modified version during fMRI. We found a significant main effect for the factor MODIFICATION_fMRI_ [*F*_(1_,_34)_ = 21.59, *p* < 0.001] indicating that after repeatedly watching modified_fMRI_ videos of a story (*M*_mod_ = 1.27 ± 0.04), participants were less likely to accept originals videos as truly experienced compared to after re-experiencing originals_fMRI_ during fMRI (*M*_*ori*_ = 1.20 ± 0.04). There were no significant interaction effects nor did we find main effects of AGENCY or PERSPECTIVE.

**FIGURE 10:**
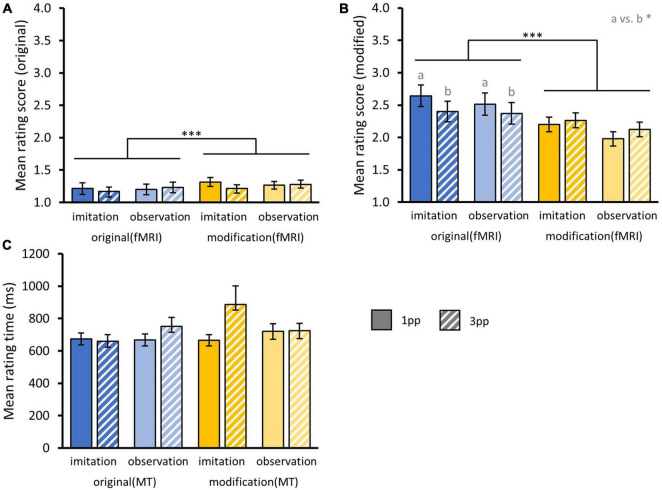
Behavioral results from post-fMRI memory test. **(A)** Mean rating scores for original and **(B)** modified videos from memory test separated by the factors AGENCY (imitation, observation) and PERSPECTIVE (1pp, 3pp) and fMRI presentation mode MODIFICATION (original, modified). Statistics: rmANOVA based on aligned ranks with *post hoc* paired *t*-tests; a vs. b = *p* < 0.05. **(C)** Overall mean rating times for correct and incorrect responses from memory test. **(A–C)** Bar charts show means and standard errors.

Second, we examined whether modified episodes were more often misclassified as known from training in the memory test after repeated presentation in the scanner. Indeed, repeated presentation of modified_fMRI_ videos (*M*_*mod*_ = 2.14 ± 0.07) led to higher acceptance of modified versions than after previously experiencing the original stories (*M*_*ori*_ = 2.48 ± 0.08), reflected in a significant main effect of MODIFICATION_fMRI_ [*F*_(1_,_34)_ = 14.94, *p* < 0.001]. We found a significant interaction effect for the factors MODIFICATION_*fMRI*_ and PERSPECTIVE [*F*_(1_,_34)_ = 5.84, *p* = 0.02] indicating that participants accepted modified stories in the memory test more often when the presentation of original stories during fMRI occurred from 3pp (*M_*ori–*3*pp*_* = 2.39 ± 0.11) than from 1pp [*M_*ori–*1*pp*_* = 2.58 ± 0.12; *t*_(34)_ = 1.86, *p* = 0.04]. This was not the case for videos presented in a modified version during fMRI [*M_*mod–*3*pp*_* = 2.19 ± 0.1, *M_*mod–*1*pp*_* = 2.09 ± 0.1; *t*_(34)_ = 0.65, *p* = 0.74], suggesting that perspective had an effect on the retrieval of the original but not the modified videos during the subsequent memory test. Note that the perspective of presenting a story during the fMRI was maintained in the memory test. There was no interaction with AGENCY nor main effects of AGENCY or PERSPECTIVE (Figures 10A,B).

In addition, we calculated a three-way rmANOVA with the factors MODIFICATION_MT_ (original_MT_, modified_MT_), PERSPECTIVE and AGENCY on rating times, including ratings for correct and false responses. Here we did not find any effect on mean rating times, but participants tended to respond faster to original versions (*M*_*ori*_ = 687.97 ms ± 21.58 ms) compared to modified ones [*M*_*mod*_ = 749.4 ms ± 34.16 ms; *F*_(1_,_34)_ = 1.33, *p* = 0.26] (Figure 10C).

Further, we calculated one-sided *t-*tests for responses and rating times for the new videos. Here, differences did not reach significance as participants showed overall low acceptance for novels. Descriptively, participants took longer to rate videos presented from 3pp [*M_*nov*–1*pp*_* = 642.05 ms ± 55.96 ms; *M_*nov–*3*pp*_* = 1002.25 ms ± 418.57 ms; *t*_(34)_ = 0.66, *p* = 0.26].

In order to examine the relationship between hippocampal activity and behavioral memory performance, correlations were computed between mean beta values in hippocampus with responses for original and modified videos during the post-fMRI memory test separated by the factors MODIFICATION (ori, mod) during fMRI and pre-fMRI AGENCY (imi, obs). As a result, there was no significant relationship between hippocampal activation and memory performance.

To summarize the behavioral results of the post-fMRI memory test, subjects took longer to rate whether they experienced a story during training when this story was presented with a slight modification. Correspondingly, videos that had already been presented in a modified version in the scanner were later more often mistaken for original episodes in this modified form. When original versions were presented in the scanner from the 3pp (compared with the 1pp), their modified versions were later more often mistaken for original episodes in the memory test.

## Discussion

Reactivating an episodic memory reinforces its stability (Karpicke and Roediger, 2008), but also allows for the integration of new information, potentially enabling adaptation to an ever-changing world (Lee et al., 2017). Performing an action during encoding and recalling it from one’s own perspective are often assumed to support memory retrieval (e.g., Hornstein and Mulligan, 2001; Marcotti and St Jacques, 2018). Therefore, we used subtle breaches of expectation in episodic cueing to test whether active self-performance (vs. passive observation) during encoding and/or first-person (vs. third-person) perspective during re-activation decrease a remembered episode’s susceptibility to modification.

Videos reminiscent of previously experienced episodes, in contrast to new videos, triggered an increased BOLD response in a network typical of episodic retrieval (Rugg and Vilberg, 2013; Jeong et al., 2015). Violating expectations of previously experienced episodes triggered an increased BOLD response to modified details in the episodic cues and descriptively prolonged RTs, as described in detail in a separate paper (Siestrup et al., 2021). In line with the here presented activation pattern during episodic retrieval, these findings confirm that subjects had successfully encoded the episodes, which provides the basis for examining the effect of agency during encoding and perspective during retrieval.

When subjects were just attentive observers and not actors themselves during the encoding of episodes, the cue modification and thus the expectation violation triggered a significantly weaker hippocampal response. While there were wide-ranging BOLD effects for 1pp (vs. 3pp) cues that occurred for new videos, these perspective effects were virtually eliminated for episodic cues. As expected, a post-fMRI memory test revealed that episodes presented in a modified version in the scanner were later more often accepted as original episodes in this modified form. Additionally, these modified versions were more often considered new in their original form, especially when presented from 3pp. Together, findings suggest that both agency during encoding and perspective of episodic cueing have a significant effect on episodic memory on the behavioral and the brain level.

### Cerebral Effects of Agency and Cueing Perspective

With regard to the effect of agency, we found a subthreshold effect (uncorrected at *p* < 0.001) of formerly imitated vs. only observed events for manipulated videos. At this level, activity increased in the left posterior PCUN, the frontopolar cortex (BA 10) of the MFC and the left hippocampus, reflecting three areas of the episodic memory network. The precuneus is involved in visuomotor imagery, action planning (Zhang and Li, 2012) and retrieval from long-term memory (Gobbini et al., 2004). Adding to these findings, our fMRI results regarding agency suggest that self-performing compared to only observing may create a stronger internal model of an episode leading to an enhanced prediction error when expectations about the specific episode are violated. As hypothesized, agency specifically affected brain responses to violated, but not to non-violated predictions (brain responses for original videos did not substantially differ on factor levels of agency). Specifically, self-performance may result in deeper encoding by enriching episodes with sensorimotor components, resulting in better retrievable memory traces than mere observation and higher sensitivity to a mismatch between stored and currently perceived information (Manzi and Nigro, 2008; Hainselin et al., 2014; Badinlou et al., 2017).

Previous research suggested that the MFC, specifically BA 10 and ACC, is involved in detecting mismatches between internal model representations and perceived information (Schiffer et al., 2012, 2013). In the present study, we found subthreshold activity in BA 10 for previously self-performed vs. only observed actions during retrieval of modified episodes. Increased activity in BA 10 may point to enhanced episodic success monitoring for previously self-performed vs. merely observed actions (Ramnani and Owen, 2004). Our results may offer an interesting starting point for future research to investigate the role of MFC subregions in processing prediction errors during episodic memory retrieval.

Following our hypotheses, we performed a ROI analysis for the left and right hippocampal complex and found a marginally significant interaction of modification and agency. Exploratory analysis revealed decreased hippocampal activity for modified episodes with a history of observation vs. imitation during encoding in left, but not right hippocampus. Though research showed stronger engagement of left hippocampus regarding the richness of actively self-encoded information (Rabin et al., 2010), our results on lateralized hippocampal activation have to be interpreted carefully as we had no hypothesis on laterality We take our results as a first indication of hippocampal contribution to expectancy violations of previously self-performed, not merely observed, aspects of an episode. We interpret reduced hippocampal activity as indicative of reduced sensitivity to the detection of episodic expectancy violations in the observation condition. When our internal model fails to predict the current perception, the hippocampus is suggested to generate a mismatch signal (Duncan et al., 2009; Long et al., 2016). Moreover, the hippocampus biases its inherent functional connectivity in response to memory prediction errors, shifting toward encoding of new information and away from retrieval of violated memory-based predictions (Bein et al., 2020). Our findings suggest that the episodic prediction error in hippocampus may be driven by the depth of encoding through a more vivid agentive state, but further validation by future research is necessary. In contrast to previous findings (e.g., Hornstein and Mulligan, 2001), a history of self-performance was ineffective on the behavioral level as self-performed episodes did not lead to better retrieval in the post-fMRI memory test.

Participants were less likely to accept original videos as truly experienced after encountering the modified version, i.e., experiencing a prediction error. While this effect was highly significant, it was small in terms of the absolute rejection rate change. Rather than modifying remembered episodes, prediction errors promoted the learning of new, alternative versions of these episodes, while only slightly alienating the original memories.

Regarding the effects of perspective during episodic memory re-activation, we could not confirm the expected BOLD increase for 1pp vs. 3pp cueing of episodes. Interestingly, contrasting novel videos with previously encountered episodes yielded highly significant effects for 1pp vs. 3pp, including increased activity in the supplementary motor area (SMA), the anterior PCUN, the postcentral gyrus, and areas belonging to the action observation network (AON; Caspers et al., 2010). Potentially, viewing new videos from the 1pp formed a sharp contrast to what the individuals lying in the scanner expected from their own perspective. Seeing an action performed by another person from one’s own perspective probably leads to a larger prediction error in the brain than watching that person from an observer perspective, as the former practically never occurs in everyday life. Our fMRI results on cueing perspective show that this strong 1pp effect can disappear when the action is part of our current expectancy repertoire. As participants experienced each story from both perspectives, they presumably encoded episodic memories in a form in which perspective was no longer critical to the generative process of episodic memory retrieval. Other studies reported differential activation for (shifting) perspectives during recall of autobiographical episodic memories (Eich et al., 2009; St Jacques et al., 2017) or during visual imagery (Grol et al., 2017). It is important to note that the type of memory reactivation in the present study differed in various ways from these. Previous studies often used the presentation of pictures or visual imagery to actively trigger episodic memories. By using pictures or verbal cues participants had to recall a scene from a certain point of view or even switch the perspective during retrieval. Thus, memory recall was dependent on the encoding perspective and the ability to mentally visualize an event from a certain perspective. Whether in our study the episodes were encoded in a perspective-neutral manner, or in two distinct variants with different perspectives, is the subject of further experiments.

### Impact of (Not) Maintaining an Episodic Story on Memory Performance

The post-fMRI memory test showed that recurrent re-activation of memories during fMRI resulted in strengthening or distorting subsequent memory performance depending on whether an original or modified video was presented in the scanner. Note that during the post-fMRI test, participants were presented with the scanner version and additionally with a counterpart version of each presented story (i.e., original_fMRI_ and modified_MT_, or modified_fMRI_ and original_MT_). After repeatedly re-experiencing a modified video during fMRI, participants showed a lower acceptance for original videos in the post-fMRI test. Note, however, that the absolute decreases in rating scores were small and acceptance rates close to ceiling. Although one could argue here that new information was acquired leading to a bias toward rejecting originals, these results ought to be interpreted with caution.

As expected, after recurrent experience of a modified video during fMRI, participants falsely accepted modified episodes more often as originals. This finding corroborates that prediction errors during episodic retrieval can lead to memory modification (Exton-McGuinness et al., 2015; Sinclair and Barense, 2018). Previous studies suggested that memory content can be overwritten, leading to a loss of previously encoded contents (Lee, 2009). Other studies rather suggest that new information is incorporated into memories, leading to biases or the formation of false memories (Schacter et al., 2011; St Jacques et al., 2013). In line with these latter studies, our results favor additional encoding of false memories, i.e., accepting a modified episode as known from training. A detailed discussion of the effects of modified episodes is provided in Siestrup et al. (2021).

### Influence of Agency and Cueing Perspective on Memory Performance

Previous research showed superior memory performance for episodes in which subjects were agents, not merely observers (Hornstein and Mulligan, 2001; Mulligan and Hornstein, 2003; Leynes and Kakadia, 2013). Considering the assumption that self-referential qualities affect the solidity and re-activation of episodic memory, we expected modified videos of previously self-performed events to be less often incorrectly accepted as known from training.

At a descriptive level, our post-fMRI results showed that previously self-performing (vs. only observing) actions led to better memory performance, irrespective of re-experiencing original or modified episodes during scanning. With regard to our multi-step actions, intensive training and visual accessibility during retrieval, the present study differs from previous studies reporting better memory after self-performance (Hornstein and Mulligan, 2001; Mulligan and Hornstein, 2003). We assume that actively performing and solely observing actions multiple times during training led to equally strong memories of experienced episodes. It has often been reported that observed actions are later remembered as self-performed, a phenomenon called “observation inflation.” In this context, it has been suggested that observing actions does also lead to the formation of motor representations, which might be why we could not find an advantage of self-performance for memory stability in the present study (Lindner et al., 2010, 2016; Leynes and Kakadia, 2013).

Episodes recalled from the 1pp perspective are generally remembered better than those recalled from the 3pp perspective (Rice and Rubin, 2009; Akhtar et al., 2017; Marcotti and St Jacques, 2018). Thus, we expected lower false memory scores (i.e., modified episodes incorrectly classified as known from training) when videos corresponded to original episodes cued from 1pp during fMRI. As expected, participants less often accepted a modified video when they encountered original counterparts in the 1pp (vs. 3pp) during fMRI, speaking in favor of a subtle perspective effect on episodic retrieval. Previous research explains such benefits by a more detailed and vivid recall of a truly experienced episode during 1pp retrieval (Rice and Rubin, 2009; Marcotti and St Jacques, 2018) and by greater availability of visual information (Libby and Eibach, 2011; Butler et al., 2016). In the present study, the latter explanation can be ruled out, since perspective of episodic cues was manipulated only by a 180° rotation. Thus, our results suggest that cueing videos from the observer perspective affected the detectability of modifications rather than the accessibility of stored visual information during retrieval. Accordingly, participants were more likely to detect changes in the post-fMRI memory test presumably because field perspective allows subjects to focus their attention more on specific features of a remembered event (Libby and Eibach, 2011). This is further corroborated by faster recognition of actions presented from 1pp vs. 3pp during fMRI.

## Conclusion

Self-referential factors, such as agency during encoding and perspective during retrieval, are suggested to shape episodic memories. The present study used episodic prediction errors to test whether or not these self-referential factors inhibit or promote the error-induced change of episodic memories, and to examine the brain processes underlying these changes. The hippocampal response was reduced to episodic prediction errors when subjects had encoded episodes only as observers. Thus, predictions derived from episodic memories based on self-performed actions might be stronger than those based on only observed actions. However, this effect was not reflected in post-fMRI memory performance. In contrast, repeated retrieval of encoded episodes from 3pp resulted in more false memories. In summary, the robust response to episodic prediction errors was subtly modulated by agency during encoding and by perspective during retrieval of episodic memory. Both factors may therefore also be relevant to the question of whether and how much is learned from episodic prediction errors. This could become the starting point for further investigations.

## Data Availability Statement

The raw data supporting the conclusions of this article will be made available by the authors, without undue reservation.

## Ethics Statement

The studies involving human participants were reviewed and approved by the Ethics Committee of the Department of Psychology, University of Münster. The patients/participants provided their written informed consent to participate in this study.

## Author Contributions

BJ, SS, NE-S, and RS contributed to conception and design of the study. BJ performed the statistical analysis and wrote the first draft of the manuscript. RS wrote sections of the manuscript and contributed with scientific support, supervision, and coordination. IT and SS provided substantial help with the data analysis. All the authors contributed to manuscript revision, read, and approved the submitted version.

## Conflict of Interest

The authors declare that the research was conducted in the absence of any commercial or financial relationships that could be construed as a potential conflict of interest.

## Publisher’s Note

All claims expressed in this article are solely those of the authors and do not necessarily represent those of their affiliated organizations, or those of the publisher, the editors and the reviewers. Any product that may be evaluated in this article, or claim that may be made by its manufacturer, is not guaranteed or endorsed by the publisher.
