# Corrigendum: Seeing What I Did (Not): Cerebral and Behavioral Effects of Agency and Perspective on Episodic Memory Re-activation

**DOI:** 10.3389/fnbeh.2022.887395

**Published:** 2022-03-24

**Authors:** Benjamin Jainta, Sophie Siestrup, Nadiya El-Sourani, Ima Trempler, Moritz F. Wurm, Markus Werning, Sen Cheng, Ricarda I. Schubotz

**Affiliations:** ^1^Department of Psychology, University of Münster, Münster, Germany; ^2^Otto Creutzfeldt Center for Cognitive and Behavioral Neuroscience, University of Münster, Münster, Germany; ^3^Center for Mind/Brain Sciences, University of Trento, Rovereto, Italy; ^4^Department of Philosophy, Ruhr University Bochum, Bochum, Germany; ^5^Institute for Neural Computation, Ruhr University Bochum, Bochum, Germany

**Keywords:** fMRI, episodic memory, perspective, agency, expectation violation, action observation, action imitation

In the original article there was an error in the Funding statement. One of the project numbers was stated as “419039274” when it should be “419038924.” The updated Funding statement is below:

## Funding

This work was funded by the German Research Foundation (Deutsche Forschungsgemeinschaft) - project numbers 419037023, 419038924, and 419037518. The funders had no role in study design, data collection, analysis and interpretation, decision to publish, or writing of the report.

The authors apologize for this error and state that this does not change the scientific conclusions of the article in any way. The original article has been updated.

## Publisher's Note

All claims expressed in this article are solely those of the authors and do not necessarily represent those of their affiliated organizations, or those of the publisher, the editors and the reviewers. Any product that may be evaluated in this article, or claim that may be made by its manufacturer, is not guaranteed or endorsed by the publisher.

