# Supplementary material for: Seeing What I Did (Not): Cerebral and Behavioral Effects of Agency and Perspective on Episodic Memory Re-activation
Source: Front Behav Neurosci. 2022 Jan 7;15:793115. doi: 10.3389/fnbeh.2021.793115 (PMC8777223; doi:10.3389/fnbeh.2021.793115)
Supplement: Supplementary file 1 [file Table_1.DOCX]

Supplementary Material

# Supplementary Figures and Tables

## Supplementary Tables

**Supplementary Table 1. Peak activations from second-level whole-brain analyses of novel videos.**

|  |  |  |  |  |  |  |
| --- | --- | --- | --- | --- | --- | --- |
| Area | H | Cluster extent (voxels) | MNI Coordinates | | | Z |
|  |  |  | x | y | z |  |
| *(nov > ori)* |  |  |  |  |  |  |
|  |  |  |  |  |  |  |
| SFS | L | 630 | -18 | 8 | 56 | 5.95 |
| IFS (BA 44) | L |  | -36 | 5 | 32 | 5.60 |
|  | R |  | 45 | 5 | 29 | 5.02 |
| IFG (BA 45) | L |  | -45 | 23 | 20 | 5.53 |
|  | R | 591 | 54 | 29 | 23 | 5.26 |
| SPL | R |  | 30 | -58 | 50 | 6.63 |
| PCUN | R | 46 | 6 | -55 | 47 | 4.86 |
| SMG | R | 99 | 42 | -34 | 41 | 4.77 |
| IPS | L | 20 | -42 | -37 | 38 | 3.95 |
| MOG | L |  | -36 | -82 | 20 | 5.21 |
| IOG ext. Into MOG, LG & pSTS | R | 1498 | 33 | -94 | -1 | 6.66 |
| FG extending into LG & pSTS | L | 1500 | -33 | -55 | -13 | 7.26 |
| FG extending into LG | R |  | 30 | -40 | -19 | 6.63 |
| aSTS | L | 8 | -51 | -10 | -13 | 4.13 |
|  | R | 121 | 51 | -10 | -19 | 5.46 |
| HC | L | 5 | -21 | -10 | -16 | 4.10 |
|  | R | 19 | 18 | -13 | -16 | 5.08 |
| Cerebellum | L | 42 | -9 | -55 | -37 | 4.97 |
|  | R | 8 | 27 | -67 | -46 | 4.42 |
|  |  |  |  |  |  |  |
|  |  |  |  |  |  |  |

*Note*: H = hemisphere; L = left; R = right; MNI = Montreal Neurological Institute; SFS = superior frontal sulcus; IFS = inferior frontal sulcus; IFG = inferior frontal gyrus; SPL = superior parietal lobe; PCUN = precuneus; SMG = supramarginal gyrus; IPS = inferior parietal sulcus; MOG = medial occipital gyrus; IOG = inferior occipital gyrus; pSTS = posterior superior temporal sulcus; aSTS = anterior superior temporal sulcus; FG = fusiform gyrus; LG = lingual gyrus; HC = hippocampus. FDR-corrected at *p* < 0.001.

## Supplementary Figures


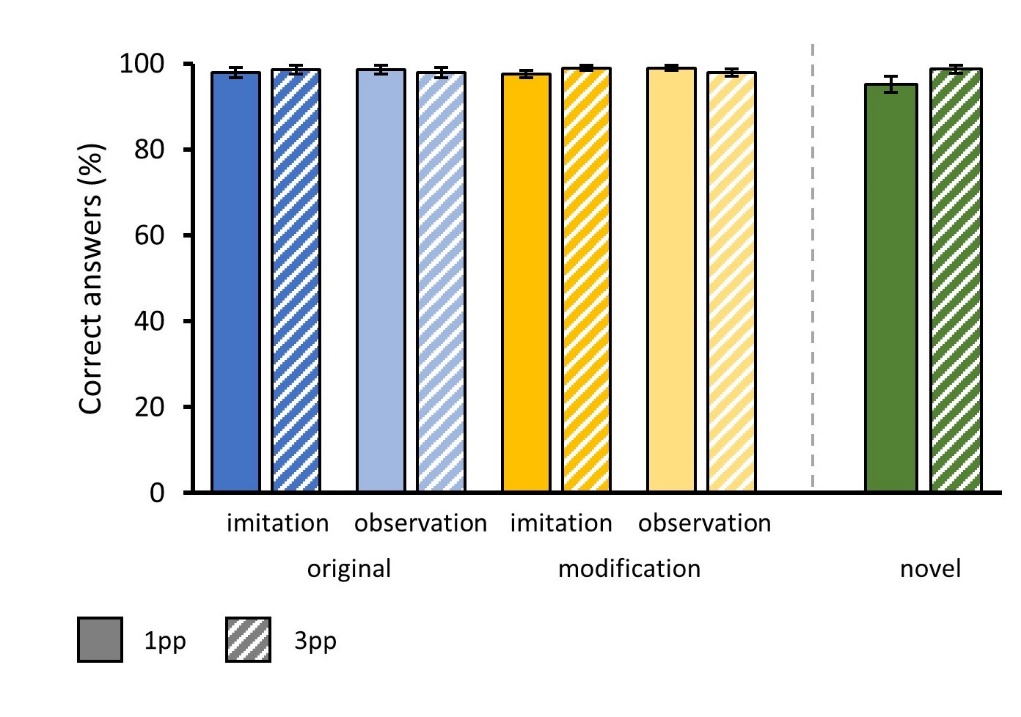


**Supplementary Figure 1. Correct response rates from fMRI task.** Mean correct response rates for original and modified videos presented during fMRI recognition task separated for the factors PERSPECTIVE (1pp, 3pp) and AGENCY (imitation, observation). Novel videos (nov) were only presented during fMRI either from 1pp or 3pp. Bar plots show means and standard errors.


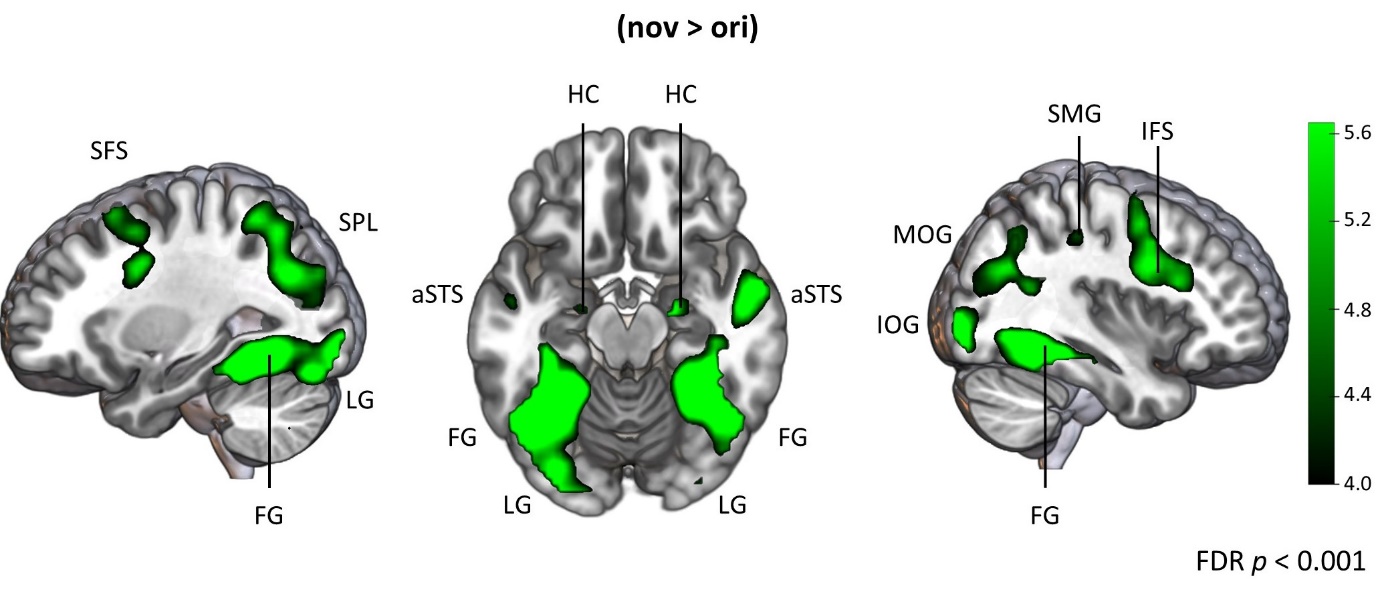


**Supplementary Figure 2. Brain activation for novels.** Brain activation for novel videos contrasted with originally encoded episodes. FDR-corrected *t*‑map (*p* < 0.001). SFS: superior frontal sulcus; SPL: superior parietal lobe; aSTS: anterior superior temporal sulcus; HC: hippocampus; FG: fusiform gyrus; LG: lingual gyrus; SMG: supramarginal gyrus; IFS: inferior frontal sulcus; MOG: medial occipital gyrus; IOG: inferior occipital gyrus.
